# Simultaneous integrated boost to lateral pelvic lymph nodes during chemoradiotherapy in high-risk rectal cancer

**DOI:** 10.1007/s00066-024-02354-z

**Published:** 2025-01-14

**Authors:** Marcel Büttner, Simon Böke, Sabrina Baumeister, Robert Bachmann, Michael Bitzer, Hans Bösmüller, Dörte Wichmann, Maximilian Niyazi, Cihan Gani

**Affiliations:** 1 Department of Radiation Oncology, University Hospital Tübingen, Hoppe-Seyler-Str. 3, 72076 Tübingen, Germany; 2 German Cancer Consortium (DKTK) Partner Site Tübingen, Tübingen, Germany; 3 German Cancer Research Center (DKFZ), Heidelberg, Germany; 4 Department of General, Visceral and Transplantation Surgery, University Hospital of Tuebingen, 72076 Tuebingen, Germany; 5 Department of Internal Medicine I, University Hospital Tübingen, Tübingen, Germany; 6 Center for Personalized Medicine, University Hospital Tübingen, Tübingen, Germany; 7 Cluster of Excellence, Image Guided and Functionally Instructed Tumor Therapies, Eberhard-Karls University, Tübingen, Germany; 8 Department of Pathology, University of Tübingen, 72072 Tübingen, Germany; 9 Department of General, Visceral and Transplantation Surgery, Interdisciplinary Endoscopy Unit, University Hospital Tübingen, Hoppe-Seyler-Straße 3, 72076 Tübingen, Germany

**Keywords:** Locally advanced rectal cancer (LARC), Dose escalation, Total mesorectal excision (TME), Preoperative chemoradiotherapy, Postoperative morbidity

## Abstract

**Background:**

Preoperative chemoradiotherapy combined with total mesorectal excision (TME) is a standard treatment for locally advanced rectal cancer (LARC). However, lateral pelvic lymph nodes (LPLNs) are often inadequately treated with standard regimens. This study examines the treatment and postoperative outcomes in LARC patients receiving a simultaneous integrated boost (SIB) for LPLNs during long-course chemoradiotherapy.

**Methods:**

This retrospective study included high-risk LARC patients (UICC, “Union Internationale Contre le Cancer”, stage III) treated with preoperative chemoradiotherapy and SIB to LPLNs. Radiotherapy was delivered to the primary tumor and elective volumes with 50.4 Gy in 28 fractions, and an SIB with a median dose of 60.2 Gy was administered to clinically positive LPLNs. TME quality and postoperative complications were assessed using MERCURY and Clavien–Dindo, respectively. Time-to-event data were analyzed according to Kaplan–Meier.

**Results:**

Between 2019 and 2023, 27 patients with high-risk LARC and LPLN metastases were treated with chemoradiotherapy. After a median follow-up of 19 months, 2‑year overall survival was 80%, disease-free survival 80%, and local control of dose-escalated lymph nodes 100%. Three patients were managed nonoperatively after a clinical complete response on endoscopy and imaging. Of the 22 patients who had surgery, only one had complications higher than Clavien–Dindo grade I; TME was graded as MERCURY I in 73%.

**Discussion and conclusion:**

The SIB approach for LPLNs in LARC is feasible, does not increase postoperative morbidity, and achieves excellent local control. This study supports the consideration of dose-escalated radiotherapy for LPLNs to address high local recurrence risks.

## Introduction

Preoperative chemoradiotherapy combined with total mesorectal excision (TME) is the standard of care for locally advanced rectal cancer (LARC) [1]. While this trimodal treatment has been highly effective in reducing local recurrences overall, it has become apparent that lateral pelvic lymph nodes (LPLNs) are insufficiently treated with standard regimens [2, 3]. For preoperative radiotherapy, the commonly used dose of 45–50 Gy is ineffective for reliably eradicating macroscopic tumor in lymph nodes. Regarding the surgical component, the extent of TME surgery includes only the removal of the rectum along with the surrounding mesorectal fat and mesorectal fascia. Although TME has significantly reduced local recurrence rates [4], lymphadenectomy (LNE) of internal or external iliac nodes is not routinely performed in most Western countries due to postoperative morbidity and functional impairments [5–7].

Approximately 50% of local recurrences (LRs) still occur in the lateral compartments in the area of the LPLNs. At the same time, 30–40% of patients with primarily enlarged LPLNs treated with chemoradiotherapy develop a lateral local recurrence (LLR) within 5 years [8, 9]. In a study of 1216 patients by Ogura et al., it was shown that malignant features in LPLN are present in approximately 17% of patients with cT3/T4 rectal cancers [2]. In the same study it was shown that in patients with LPLNs with evidence of malignancy, trimodality treatment with radiotherapy and TME with LPLN dissection led to a significantly lower 5‑year LLR of 5.7% compared to 25.6% in patients with radiotherapy and TME only [2].

Previous studies have suggested that a strategy to address this limitation is to increase the radiotherapy dose for LPLNs [10, 11]. In the present study, we report the treatment and postoperative outcomes of patients with locally advanced rectal cancer who received a simultaneous integrated boost (SIB) to LPLNs within the context of long-course chemoradiotherapy.

## Methods

### Inclusion criteria

This retrospective study included patients with LARC (UICC, “Union Internationale Contre le Cancer” stage III) who were treated with preoperative chemoradiotherapy and SIB to at least one LPLN. All patients had magnetic resonance imaging (MRI) of the pelvis and computed tomography of the chest and abdomen as staging procedures.

Lateral pelvic lymph nodes were considered affected if they met a least two of the following criteria: round shape, short axis diameter of more than 7 mm, and mixed signal intensity on MRI (analogous to Ogura et al. 2019, Kim et al. 2008, and Li et al. [2, 11, 12]).

### Treatment protocols

**Fig. 1:**
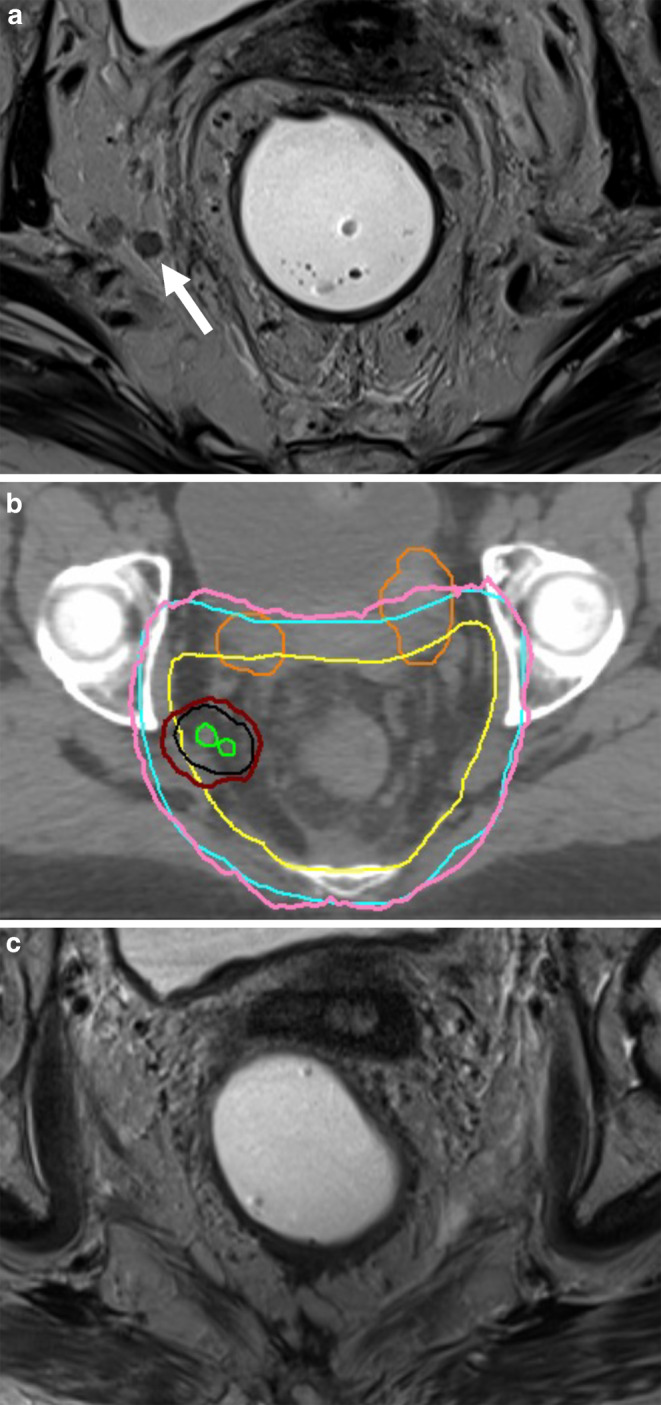
**a** Example of a female patient who presented with two round-shaped lateral pelvic lymph nodes (white arrow; **b**). The lymph nodes (in green; corresponding planning target volume [PTV] in black) were treated with 61.6 Gy in 28 fractions; the red line shows the 95% isodose relative to the 61.6 Gy volume. The pink line indicates the 95% isodose relative to the 50.4 Gy PTV in blue prescribed to the mesorectum and elective nodal volumes. Bowel loops are segmented in orange. **c** The patient achieved a clinical complete response at all tumor sites (primary, mesorectal lymph nodes, and lateral pelvic lymph nodes) and was managed nonoperatively

Radiotherapy was planned as intensity-modulated radiotherapy (IMRT) on a linear accelerator with daily cone-beam computed tomography (CT)-based image guidance in all cases. IMRT planning was performed on CT scans with 3 mm slice thickness. Patients were positioned in supine position as per our institutional standard. Target volume definition for the primary tumor and the local lymphatics was according to international guidelines [13]. For small bowel and the bladder, a maximum point dose of 56 and 62 Gy, respectively, was accepted. All patients received chemoradiotherapy at the Department of Radiation Oncology of the University Hospital Tübingen, Germany. The primary tumor, the mesorectum, and lateral pelvic lymph node stations received 50.4 Gy in 28 fractions, and positive LPLNs received a median dose of 60.2 Gy (interquartile range 60.2–61.6 Gy). A case example with positive LPLNs and the treatment plan is shown in Fig. 1a, b. Concomitant chemotherapy was applied as 5‑FU alone or combined with oxaliplatin, as previously reported [14]. Consolidative chemotherapy with FOLFOX or CAPOX was considered if organ preservation was intended or to improve disease-free survival, after the publication of two randomized phase III studies proved the benefit of total neoadjuvant therapy (TNT) [15, 16].

Total mesorectal excision was performed according to standard techniques. Abdominoperineal extirpation was indicated if the distal end of the tumor was within 5 mm of the dentate line after radiochemotherapy. Patients were considered for nonoperative management when a clinical complete response (cCR) was seen after chemoradiotherapy. A cCR was defined as the absence of any residual finding beyond a white scar, telangiectasis, or a small ulcer on endoscopy. In this scenario, MRI was used to evaluate previously suspicious lymph nodes. Lymph nodes were considered negative if they were smaller than 5 mm in the short axis.

Total mesorectal excision quality was evaluated according the MERCURY grading system [17], and postoperative complications were evaluated according to Clavien–Dindo [18].

Continuous data are reported with the median and interquartile range (IQR). Toxicity was assessed according to the Common Terminology of Adverse Events (CTCAE). Time-to-event data were calculated according to Kaplan–Meier. Overall survival (OS) was the time from primary diagnosis until death. Disease-free survival (DFS) was calculated from primary diagnosis until death or any recurrence. For local control (LC), progression of the dose-escalated lesion on an imaging study was considered an event. Median follow-up was determined according to the inverse Kaplan–Meier method. For group comparisons, the log-rank test was used. Statistical analyses were conducted in SPSS (SPSS 26, IBM, Armonk, NY, USA) and R (version 4.0.3; R Foundation, Vienna, Austria).

The study was approved by the ethics committee of the Medical Faculty of Tübingen, Germany (approval ID: 733/2015B01).

## Results

Between 2019 and 2023, 27 patients met the inclusion criteria. Patient- and tumor-related parameters are shown in Table 1. The mesorectal fascia was affected in 22 (81%) patients, and six tumors (22%) showed extramural venous invasion (EMVI). The median volumes of the primary tumor, the clinical target volume of the pelvis, and the lateral pelvic lymph nodes were 56 ccm (IQR 24.8–106.8), 829 ccm (IQR 629.0–975.9), and 1.8 ccm (IQR 0.9–4.5), respectively. The planning target volume for the pelvic target volume and the lymph node SIB were 1370 ccm (IQR 1208.4–1728.8) and 8.4 ccm (IQR 4.8–23.1), respectively. Patients received concomitant chemoradiotherapy at full dose. No CTCAE grade 3 or 4 toxicity probably related to the SIB was observed. In addition to 5‑FU, 15 patients (56%) were treated with oxaliplatin during radiotherapy, as reported previously [14]. Twelve patients (44%) received a maximum of four cycles of consolidation chemotherapy with CAPOX or six cycles of FOLFOX. After restaging, three patients (11%) were classified as having a clinical complete response and omitted surgery; all three patients have completed at least 2 years of follow-up and are disease free. Two other patients did not undergo surgery. One of these two patients was diagnosed with diffuse metastatic disease after completion of chemoradiotherapy. The other patient died after a fulminant upper gastrointestinal bleed. The remaining 22 patients (81%) underwent surgery after a median time of 114 days (IQR 55–155) after completion of radiotherapy. During the postoperative course, 21 of 22 patients (95%) had no more than Clavien–Dindo I complications. One patient had anastomotic insufficiency requiring endovac treatment. Surgical and pathological outcome parameters are listed in Table 2.

**Table 1:** Patient characteristics, tumor-related parameters, and treatment regimens

***Number of patients***	27	
***Sex***		
Female	7	(26%)
Male	20	(74%)
**Median age at initial diagnosis (IQR)**	63 (54–71)	
***Tumor-related parameters***		
**Tumor localization**		
0–6 cm	9	(33%)
6.1–12 cm	18	(67%)
**Stage and risk factors**		
**T category**		
cT2	1	(4%)
cT3	17	(63%)
cT4a	5	(19%)
cT4b	4	(15%)
**N category**		
cN1	10	(37%)
cN2	17	(63%)
**UICC**		
Stage III	27	(100%)
**Further risk factors**		
LPLN+	27	(100%)
MRF+	22	(81%)
EMVI+	6	(22%)
***Treatment regimens***		
**Radiotherapy**		
Long-course CRT (50.4 Gy in 28 fractions)	27	(100%)
Median SIB dose to LPLNs in Gy (IQR)	60.2 (60.2–61.6)	
**Concomitant chemotherapy**		
5‑FU	11	(41%)
5‑FU and oxaliplatin	15	(56%)
Capecitabine and oxaliplatin	1	(4%)
**Post-CRT treatment**		
Surgery	22	(81%)
*Abdominoperineal resection*	6	(22%)
*Low anterior resection*	16	(59%)
Watch and wait	3	(11%)
Death or progression after CRT	2	(7%)
Median time between CRT to resection in days (IQR)	114 (54.5–155.25)	
**Consolidation chemotherapy**		
4 cycles CAPOX or 6 cycles FOLFOX	12	(44%)

*IQR* interquartile range, *UICC* Union Internationale Contre le Cancer, *LPLN+* affection of lateral pelvic lymph nodes, *MRF*+ affection of the mesorectal fascia,* EMVI*+ extramural venous invasion, *CRT* chemoradiotherapy, *SIB* simultaneous integrated boost

Percentages might not sum up to 100% due to rounding

**Table 2:** Pathological outcome parameters and postoperative course

*Patients resected*	22	
*Pathological complete remission (pCR)*	3	(14%)
*Pathological T category after chemoradiotherapy*		
ypT0	3	(14%)
ypT1	3	(14%)
ypT2	6	(27%)
ypT3	6	(27%)
ypT4a	3	(14%)
ypT4b	1	(5%)
*Pathological N category after chemoradiotherapy*		
ypN0	16	(73%)
ypN1	3	(14%)
ypN2	3	(14%)
*L category*		
L0	19	(86%)
L1	3	(14%)
*V category*		
V0	22	(100%)
*Pn category*		
Pn0	19	(86%)
Pn1	3	(14%)
*Tumor regression*		
Dworak 1	4	(18%)
Dworak 2	7	(32%)
Dworak 3	5	(23%)
Dworak 4	3	(14%)
Missing	3	(14%)
*Completeness of TME*		
MERCURY 1	16	(73%)
MERCURY 2	3	(14%)
MERCURY 3	2	(9%)
Missing	1	(5%)
*Postoperative morbidity*		
Normal postoperative course	18	(82%)
Clavien–Dindo I^a^	2	(9%)
Clavien–Dindo III^b^	1	(5%)
Missing	1	(5%)

Percentages might not sum up to 100% due to rounding.

^a^Clavien–Dindo I complications were transient urinary retention and delayed bowel passage

^b^Clavien–Dindo III complication was an anastomotic insufficiency requiring endovac treatment

**Fig. 2:**
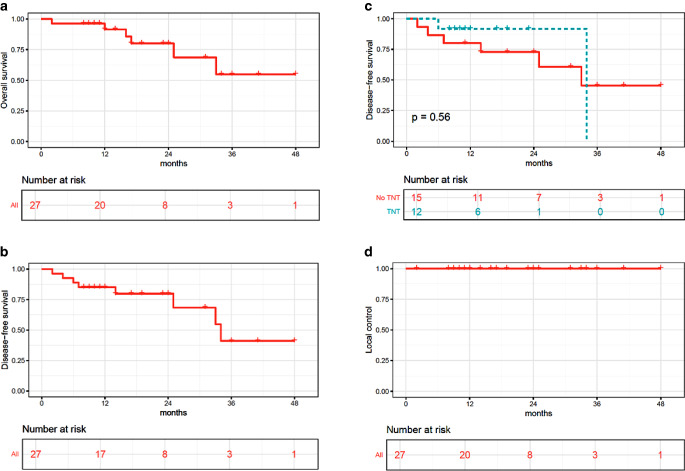
Kaplan–Meier curves over 2 years with **a** overall survival, **b** disease-free survival (DFS) of the entire cohort, **c** DFS divided into a cohort with total neoadjuvant therapy (TNT) and without TNT, and **d** local control of lymph nodes treated with dose escalation

After a median follow-up of 19 months, six patients had died, resulting in a 2-year Kaplan–Meier estimation for overall survival of 80% (Fig. 2a). Two-year DFS for the entire cohort was 80% (Fig. 2b). Two-year DFS for patients who had received consolidation chemotherapy was 92%, whereas it was 73% for those who did not receive consolidation chemotherapy (Fig. 2c, *p* = n. s.). Local control in lymph nodes treated with dose escalation was 100% (Fig. 2d). An example is shown in Fig. 1c. Overall, three patients developed local and distant recurrence and three patients only distant failure.

## Discussion

The optimal management of LPLNs in rectal cancer is an ongoing debate [8]. While there is a growing body of evidence for dose escalation to the primary tumor, the literature on dose escalation for LPLNs is limited, and prospective data are lacking [19, 20]. In particular, the impact of dose-escalated radiotherapy on postoperative morbidity and TME quality has not been reported so far. In the present study, we were able to show the feasibility of an SIB approach to clinically positive lateral pelvic lymph nodes in rectal cancer. The median dose of 60.2 Gy to LPLNs was chosen in analogy to commonly used doses for positive lymph nodes in, for example, prostate cancer, which have been proven to be feasible with no evidence of increased toxicity [21]. The latter is reflected by only one patient with Clavien–Dindo grade III complications, which is in line with previous reports [17]. Furthermore, despite the higher dose to the LPLNs, the rate of complete TME according to MERCURY was comparable with published data for patients without dose escalation [22, 23]. This aspect is of particular relevance since a strong correlation between the completeness of TME and local and distant recurrences has been reported [24]. Besides the cN2 or cT4 category, MRF positivity, and EMVI, LPLN positivity was one of the high-risk criteria that qualified for inclusion in the RAPIDO study that tested total neoadjuvant therapy against long-course preoperative chemoradiotherapy with optional adjuvant chemotherapy. The study showed a significant benefit of TNT in terms of disease-related treatment failures [15]. In a subsequent report, LPLN positivity was an independent risk factor for local recurrence in multivariate analysis irrespective of the treatment arm [25]. This underscores the importance of treatment alternatives specifically addressing this source of local recurrences. In our cohort, LC was excellent, with no recurrences originating from dose-escalated lymph nodes, which is in concordance with previous reports on dose escalation to LPLNs [11, 26]. The cohort in our study is composed of patients at a very high risk of local or distant recurrence. For instance, 81% showed MRF positivity, 63% had cN2 tumors, and 33% cT4 tumors, which are higher rates than in most other published cohorts treated with dose-escalated radiotherapy to LPLNs [11, 26]. This likely explains the lower rates of DFS and OS compared with the available literature [11, 26] and supports the application of consolidation chemotherapy in these patients.

Our study does have some limitations. While the focus of our study was on feasibility and early outcome parameters, the short follow-up is a limitation and hampers comparisons with the existing literature. Further limitations are the retrospective design and the small sample size. MRI is the diagnostic tool of choice for staging of rectal cancer and has proven to be very reliable in predicting MRF positivity and T category [27]. However, nodal staging in rectal cancer remains challenging, and using size as the only imaging feature has been shown to be of limited sensitivity [28]. However, sensitivity does improve if morphological aspects such as shape, border, and signal heterogeneity are included in the assessment, as done in our study [29]. Nonoperative management in case of a clinical complete response has emerged as an alternative to surgery over recent years [30–32]. A subgroup of patients in our study has achieved a clinical complete response and remained free of regrowth after at least 2 years of follow-up. This finding is of importance, as it shows that the presence of LPLNs should not be a criterion to preclude patients from nonoperative management.

## Conclusion

Overall, the current study shows that dose-escalated radiotherapy of LPLNs using an SIB is feasible and does not cause increased postoperative morbidity in a patient cohort with an overall poor prognosis. Furthermore, the high rate of distant recurrences supports the use of consolidation chemotherapy in this group of patients with a poor prognosis.
